# Automatic NMR-Based Identification of Chemical Reaction Types in Mixtures of Co-Occurring Reactions

**DOI:** 10.1371/journal.pone.0088499

**Published:** 2014-02-13

**Authors:** Diogo A. R. S. Latino, João Aires-de-Sousa

**Affiliations:** 1 CQFB, REQUIMTE, Departamento de Química, Faculdade de Ciências e Tecnologia, Universidade Nova de Lisboa, Caparica, Portugal; 2 CCMM, Departamento de Química e Bioquímica, Faculdade de Ciências, Universidade de Lisboa, Lisboa, Portugal; University of Sydney, Australia

## Abstract

The combination of chemoinformatics approaches with NMR techniques and the increasing availability of data allow the resolution of problems far beyond the original application of NMR in structure elucidation/verification. The diversity of applications can range from process monitoring, metabolic profiling, authentication of products, to quality control. An application related to the automatic analysis of complex mixtures concerns mixtures of chemical reactions. We encoded mixtures of chemical reactions with the difference between the ^1^H NMR spectra of the products and the reactants. All the signals arising from all the reactants of the co-occurring reactions were taken together (a simulated spectrum of the mixture of reactants) and the same was done for products. The difference spectrum is taken as the representation of the mixture of chemical reactions. A data set of 181 chemical reactions was used, each reaction manually assigned to one of 6 types. From this dataset, we simulated mixtures where two reactions of different types would occur simultaneously. Automatic learning methods were trained to classify the reactions occurring in a mixture from the ^1^H NMR-based descriptor of the mixture. Unsupervised learning methods (self-organizing maps) produced a reasonable clustering of the mixtures by reaction type, and allowed the correct classification of 80% and 63% of the mixtures in two independent test sets of different similarity to the training set. With random forests (RF), the percentage of correct classifications was increased to 99% and 80% for the same test sets. The RF probability associated to the predictions yielded a robust indication of their reliability. This study demonstrates the possibility of applying machine learning methods to automatically identify types of co-occurring chemical reactions from NMR data. Using no explicit structural information about the reactions participants, reaction elucidation is performed without structure elucidation of the molecules in the mixtures.

## Introduction

As the chemical composition of complex mixtures change with time, so do their NMR spectra. The interpretation of spectra modifications in terms of chemical reactions taking place has the potential to elucidate underlying chemical phenomena. Machine learning can extract knowledge from complicated databases of experimental observations, to recognize patterns in new situations. Automatic reaction identification can be useful in many different applications, e.g. to study chemical stabilities and aging of consumer/industrial products, to monitor biotechnological processes, or to assess the function of new enzymes in a pool of possible substrates.

Patterns of NMR changes are expected to be associated with types of reactions, because the atoms near the reaction center have their environment modified – and their NMR properties altered –, whereas the substructures of the reactants that are far from the reaction center will be mostly unchanged. Additionally, NMR spectra are sensitive to changes in the 3D environment of atoms, which may be observed even in substructures topologically distant from the reaction center and can be typical of certain reactions. Machine learning techniques should be able to recognize types of chemical reactions from NMR changes, even when more than one reaction occur simultaneously.

The processing of NMR spectra with chemometric and machine learning techniques is extensively used in the analysis of complex mixtures [1], notably in metabonomics [2]. Examples include the classification of lung carcinoma cell lines [3], the classification of human saliva according to treatment with an oral rinse formulation, or donor [4], [5], the analysis of human plasma to study metabolic changes caused by diet [6], the assessment of how concentration patterns of hydrophilic and lipophilic tissue metabolites describe different stages of breast tumor malignancy [7], or the identification of lipoprotein subclasses in plasma samples [8].

Other areas where NMR-based machine learning methods have been applied are the authentication of products [9]–[10], monitoring of enzymatic reactions [11], or assessment of drug toxicity [12]. Alonso-Salces et. al. used pattern recognition techniques (LDA, PLS-DA, SIMCA, and CART) for the geographical characterization of virgin olive oils based on the ^1^H NMR fingerprint of the unsaponifiable matter [9]. Aursand and co-workers have reported the ability of Kohonen Self-Organizing Maps (Kohonen SOMs) and generative topographic mapping to discriminate ^13^C NMR spectra of different commercial fish oil-related health food products concerning the nature, composition, refinement, and/or adulteration [10].

NMR techniques are well established for monitoring chemical and enzymatic reactions, industrial processes, and for the elucidation of reaction mechanisms. A few examples are mentioned next that also illustrate the current development of new instruments specifically designed for reaction analysis. Ballard et al. [13] used quantitative NMR to measure the concentration of carbamates over time, in order to study the chemical reaction of CO_2_ with mixtures of amines. Shey et al. [14] monitored polymer-supported reactions with conventional ^1^H NMR spectroscopy during a liquid-phase combinatorial synthesis. Kalelkar and co-workers applied SOMs to analyse NMR spectra from combinatorial parallel synthesis [15]. Bernstein et al. [16] described an apparatus consisting in a reactor coupled with an NMR flow cell; more recently an NMR flow cell based on a standard 5 mm NMR tube was presented that can be used for homogeneous and heterogeneous reactions [17]. Gomez et al. [18] presented a nanolitre NMR spectroscopy microfluidic chip hyphenated to a continuous flow microlitre-microwave irradiation set-up, for on-line monitoring. The method was also applied for rapid optimization of reaction conditions. Mix et al. [19] developed a double-chamber NMR tube – differently of others, this apparatus provides the full control of the temperature over the range from −80 to 130°C. Foley et al. [20] developed the ReactNMR method for reaction monitoring and *in situ* characterization of reaction intermediates, assisting in mechanism elucidation and in the characterization of complex reaction mixtures.

Our lab has previously shown that Kohonen Self-Organizing Maps (Kohonen SOMs) and random forests can classify individual reactions from the difference between the ^1^H NMR spectrum of the products and the reactants [21]. The obtained models can then be applied in new situations, even if the structures of the reactants and products are unknown, but their ^1^H NMR spectra are available.

Here we present an extension of this approach to mixtures of reactions. As before, machine learning methods received as input the difference between the ^1^H NMR spectra of the products and the reactants – but now the products of two reactions of different classes are taken together, as well as the reactants. This simulates a situation in which two reactions occur simultaneously. The SPINUS program [22]–[24] was used to estimate ^1^H NMR chemical shifts from the molecular structure, and the chemical shifts were fuzzified to tolerate small variations. Three machine learning methods were explored that differ in the type of learning. Kohonen SOMs are trained with unsupervised learning (competitive learning), counter-propagation neural networks (CPNN) use semi-supervised learning, and random forests (RF) use supervised learning. A dataset of 181 photochemical cycloadditions manually assigned into six types was used to simulate 12,421 mixtures of two reactions. The machine learning algorithms were given the task of predicting the types of reactions, in a simulated situation where two reactions of different types occur simultaneously, from the simulated ^1^H NMR spectra of the reactants and products.

## Methods

The experiments here described involve three main steps: a) the generation of a reaction descriptor from the simulated ^1^H NMR spectra of the products and reactants; b) the generation of the simulated mixtures of two reactions from the NMR reaction descriptors; c) the development of classification models for mixtures of reactions taking as input ^1^H NMR data.

### Data Sets of Reactions

**Figure 1:**
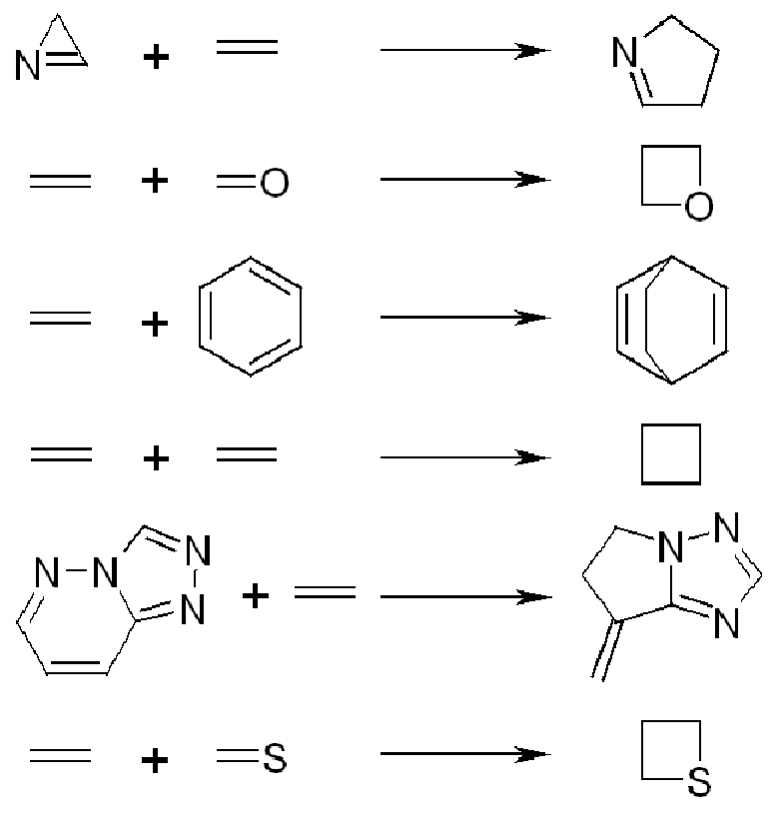
Types of photochemical reactions (from top): [3+2] photocycloaddition of azirines to C = C, [2+2] photocycloaddition of C = C to C = O, [4+2] and [4+4] photocycloaddition of olefins to carbon-only aromatic rings, [2+2] photocycloaddition of C = C to C = C, [3+2] photocycloaddition of s-triazolo[4,3-b]pyridazine to C = C, and [2+2] photocycloaddition of C = C to C = S.

A data set of 181 photochemical reactions, involving two reactants and one product (bearing at least one hydrogen atom covalently bonded to a carbon atom) was extracted from the SPRESI database (InfoChem GmbH, Munich, Germany). The reactions were manually assigned into six types (Figure 1): [3+2] photocycloaddition of azirines to C = C (20 reactions), [2+2] photocycloaddition of C = C to C = O (31 reactions), [4+2] and [4+4] photocycloaddition of olefins to carbon-only aromatic rings (20 reactions), [2+2] photocycloaddition of C = C to C = C (73 reactions), [3+2] photocycloaddition of s-triazolo[4,3-b]pyridazine to C = C (10 reactions), and [2+2] photocycloaddition of C = C to C = S (27 reactions). [21]

We simulated all possible mixtures of two reactions (belonging to different types) from the data set of 181 reactions. For example, the 20 *[3+2] photocycloaddition of azirines to C = C* and the 31 *[2+2] photocycloaddition of C = C to C = O* yield 620 mixtures of reactions of class A. In the following combination the 20 *[3+2] photocycloaddition of azirines to C = C* and the 20 *[4+2] and [4+4] photocycloaddition of olefins to carbon-only aromatic rings* yield 400 mixtures of reactions of class B, and so on until all possible combinations of reactions types were simulated. The final data set of mixtures of reactions consists in 12421 mixtures. From this data set, 8280 mixtures were randomly selected to the training set and the remaining 4141 were used as a test set, Partition 1. Figure 1 and Table 1 show the types of reactions and the number of reactions by type. Table 2 indicates the resulting number of mixtures.

**Table 1:** Number of reactions by reaction type and partition to be used to generate training and test sets of Partition 2 of mixtures of reactions.

Types of Reactions	Number of Reactions	For Partition 2*
[3+2] photocycloaddition of azirines to C = C	20	16/4
[2+2] photocycloaddition of C = C to C = O	31	23/8
[4+2] and [4+4] photocycloaddition of olefins to carbon-only aromatic rings	20	16/4
[2+2] photocycloaddition of C = C to C = C	73	56/17
[3+2] photocycloaddition of s-triazolo[4,3-b]pyridazine to C = C	10	8/2
[2+2] photocycloaddition of C = C to C = S	27	21/6
Total	181	140/41

*Number of reactions in the training/test set to be used to generate Partition 2 of mixtures of reactions.

**Table 2:** Number of reaction mixtures in each mixture class (mixture of two reactions of different types) for the two partitions of the data set.

Class of mixture	Reaction 1	Reaction 2	Partition 1*	Partition 2*
A	[3+2] photocycloaddition of azirines to C = C	[2+2] photocycloaddition of C = C to C = O	413/207	368/32
B	[3+2] photocycloaddition of azirines to C = C	[4+2] and [4+4] photocycloaddition of olefins tocarbon-only aromatic rings	267/133	256/16
C	[3+2] photocycloaddition of azirines to C = C	[2+2] photocycloaddition of C = C to C = C	975/487	896/68
D	[3+2] photocycloaddition of azirines to C = C	[3+2] photocycloaddition of s-triazolo[4,3-b]pyridazineto C = C	132/67	128/8
E	[3+2] photocycloaddition of azirines to C = C	[2+2] photocycloaddition of C = C to C = S	360/180	352/20
F	[2+2] photocycloaddition of C = C to C = O	[4+2] and [4+4] photocycloaddition of olefins tocarbon-only aromatic rings	413/206	368/32
G	[2+2] photocycloaddition of C = C to C = O	[2+2] photocycloaddition of C = C to C = C	1510/754	1288/136
H	[2+2] photocycloaddition of C = C to C = O	[3+2] photocycloaddition of s-triazolo[4,3-b]pyridazineto C = C	206/104	184/16
I	[2+2] photocycloaddition of C = C to C = O	[2+2] photocycloaddition of C = C to C = S	558/279	506/40
J	[4+2] and [4+4] photocycloaddition of olefinsto carbon-only aromatic rings	[2+2] photocycloaddition of C = Cto C = C	974/486	896/68
K	[4+2] and [4+4] photocycloaddition of olefinsto carbon-only aromatic rings	[3+2] photocycloaddition of s-triazolo[4,3-b]pyridazineto C = C	133/67	127/8
L	[4+2] and [4+4] photocycloaddition of olefinsto carbon-only aromatic rings	[2+2] photocycloaddition of C = C to C = S	360/180	353/20
M	[2+2] photocycloaddition of C = C to C = C	[3+2] photocycloaddition of s-triazolo[4,3-b]pyridazineto C = C	498/250	448/34
N	[2+2] photocycloaddition of C = C to C = C	[2+2] photocycloaddition of C = C to C = S	1302/651	1232/85
O	[3+2] photocycloaddition of s-triazolo[4,3-b]pyridazine to C = C	[2+2] photocycloaddition of C = C to C = S	180/90	176/10

*Number of reactions in the training/test sets.

Another more challenging partition of the data set was also used in which the data set of 181 reactions was randomly partitioned into subsets of 140 and 41 reactions, assuring that both sets cover the whole range of reactions, (see Table 1 for training and test set partition by reaction type) and the combinations were generated within each data set. For example the 16 *[3+2] photocycloaddition of azirines to C = C* and the 23 *[2+2] photocycloaddition of C = C to C = O* yield 368 mixtures of reactions of class A for the training set then, in the following combination, the 16 *[3+2] photocycloaddition of azirines to C = C* and the 16 *[4+2] and [4+4] photocycloaddition of olefins to carbon-only aromatic rings* yield 256 mixtures of reactions of class B for the training set of Partition 2, and so on until all possible combinations of reactions were simulated. The same was performed for the test set. The larger subset (7578 mixtures) was used as a training set and the smaller (593 mixtures) as a test set consisting in Partition 2 (see Figure 1 and Table 1 for types of reactions and number of reactions by type, and Table 2 for the resulting number of mixtures). Table 2 shows the constitution of each data set and the labels used in the experiments with Kohonen SOMs. Mixture classes (A to O) correspond to combinations of two reaction types. It is to emphasize that a 15-class classification problem like this (15 different mixtures of reactions) is a challenging modelling problem even using supervised learning techniques.

### ^11B1^H NMR Spectra of Reactants and Products

^1^H NMR chemical shifts were predicted by the SPINUS program (v2) [22]–[24] from the molecular structures of the reactants and products. Only hydrogen atoms bonded to carbon atoms were predicted. The predicted chemical shifts were fuzzified with a triangular function and widths 0.1 ppm at each side of the chemical shift, which approximate the observed mean absolute error of SPINUS predictions (0.2–0.3 ppm). [22]

### ^1^H NMR Spectra of Mixtures before and after the Reactions

All the signals, integrating proportionally to the number of protons, arising from all reactants of one reaction were taken together (spectrum of the reactants). The spectrum of the reactants was subtracted from the spectrum of the product. This is the difference between the spectra after and before the reaction, assuming full conversion.

The difference spectrum (“reaction spectrum”) was binned in the range 0–12 ppm using 0.1 ppm wide intervals resulting in 120 variables (each variable integrating the intensities within an interval of 0.1 ppm). Experiments concerning the optimization of the binning procedure and integration of the intensities and their relation with the mean absolute error was performed in a previous publication [21].

For a mixture of two reactions, the reactions spectra are summed. The result corresponds to the difference of the spectra before (only reactants) and after (only products) the two reactions occur. Simultaneousness of the two reactions is assumed. Unless otherwise specified, the two reactions of each mixture were simulated in a 1∶1 ratio and with full conversion.

In this way, we generate an NMR reaction descriptor for each reaction mixture of the data set, which is used as the input to the machine learning techniques.

### Machine Learning Methods

#### Kohonen Self-Organizing Maps (Kohonen SOMs) [25], [26]

Kohonen SOMs learn by unsupervised training, distributing objects through a grid of so-called neurons, on the basis of the objects’ features. This is an unsupervised method that projects multidimensional objects into a 2D surface (a map). SOM can reveal similarities between objects, mapped into the same or neighbor neurons. Each neuron of the map contains as many elements (weights) as the number of input variables (objects features). Before the training starts, the weights take random values. During the training, each individual object is mapped into the neuron with the most similar weights compared to its features (shortest Euclidean distance between weights and input). This winning neuron is excited (or activated), and its weights are corrected to make them even more similar to the object features. The neurons in its neighborhood also have their weights adjusted. The extent of adjustment depends on the topological distance to the winning neuron – the closer a neuron is to the winning neuron the more it is adjusted – and on the stage of training. The objects of the training set are iteratively fed to the map, and the weights corrected, until a pre-defined number of cycles is attained. A trained Kohonen SOM reveals similarities between objects of a data set in the sense that similar objects are mapped into the same or closely adjacent neurons.

In the investigations described here, the input variables are the 120 NMR reaction descriptors derived from the spectra of the reactants and products of two reactions. SOMs with toroidal topology and dimension 49×49 were trained and tested using the two different partitions of the data set. The maximum size was chosen such that the number of mixtures of reactions was at least twice the number of neurons. The toroidal topology means that neurons occupy the surface of a torus, so that all neurons have 8 neighbors – in the 2D representation of the map the neurons at the left edge are neighbors of those at the right edge, and the same happens for those at the top and bottom edges. After the training, each neuron is labeled (colored) according to the classes of reaction mixtures that activate it (see Table 1), which facilitates visualization, and enables the classification of new reaction mixtures.

Training was performed by using a linear decreasing triangular scaling function with an initial learning rate of 0.1. The weights were initialized with random numbers that are calculated using the mean and the standard deviation of each variable in the input data set. For the selection of the winning neuron, the minimum Euclidean distance between the input vector and neuron weights was used. The training was performed over 50–100 cycles, with the learning span and the learning rate linearly decreasing until zero. These parameters appeared as a reasonable balance between network stability and computation time. Kohonen SOM were implemented with in-house-developed software based on JATOON Java applets. [27] To overcome fluctuations induced by the random factors influencing the training, five or ten independent SOM were trained with the same objects, generating an ensemble of SOM. Ensemble predictions were obtained for new objects by majority vote of the individual SOMs.

#### Counter-Propagation Neural Networks (CPNNs) [26]

A Counter-Propagation Neural Networks incorporate a Kohonen SOM linked to a second layer of neurons (output layer) that acts as a look-up table and stores output data (the classification of the mixture of reactions). The CPNN method is considered a semi-supervised technique. During the training, the winning neuron is determined exclusively on the basis of the Kohonen layer (input layer), but the weights of the corresponding output neuron are adjusted to become closer to the output values of the object – semi-supervised learning. After the training, the CPNN can produce an output for an object – the winning neuron is chosen and the corresponding weights in the output layer are taken as the prediction.

In this work, the types of the mixture reactions were encoded into a vector (output) with dimension six (the number of reaction types). The two components of the vector corresponding to the types of the reactions present in the mixture take the value one, the others take the value zero. After the training, when working in prediction mode, CPNN produce a six-values output for a reaction mixture, which is interpreted as a prediction of the two types obtaining the highest values.

Software and training settings were the same as in the experiments with Kohonen SOMs. Ensembles of five or ten independent CPNN were trained, and predictions were obtained by majority vote of the individual maps.

#### Random Forests (RF) [28], [29]

A random forest is an ensemble of unpruned classification trees created by using bootstrap samples of the training data and random subsets of variables to define the best split at each node. It is a high-dimensional nonparametric method that works well on large numbers of variables. The predictions are made by majority voting of the individual trees. The performance is internally assessed with the prediction error for the objects left out in the bootstrap procedure (out-of-bag estimation, OOB). Here, RFs were grown with the R program version 2.0.1, [30] using the randomForest library, [31] and were used to classify the reactions present in a mixture of reactions on the basis of NMR reaction descriptors. The models were built to classify objects (mixtures of reactions) according to the 15 classes of mixtures (classes A to O, Table 1). The number of trees in the forest was set to 1000, and the number of variables tested for each split was set to default (square root of the number of variables). The voting system of a RF allows the association of a probability to each prediction that reflects the percentage of votes obtained by the winning class. This probability was investigated as a measure of reliability.

## Results and Discussion

Previous to this work, the chemical shifts predicted by SPINUS had been validated for a subset of reactants and products in our data set of reactions, for which experimental chemical shifts were available [21]. A mean absolute error (MAE) of 0.24 ppm was obtained for the 349 chemical shifts of the subset, which was similar to earlier tests [23].

The following subsections present results concerning the ability of various machine learning methods (including unsupervised and supervised learning) to recognize patterns of changes in the ^1^H NMR spectra corresponding to types of reactions, when two reactions occur simultaneously. Experiments were performed with two different partitions of the data set.

### Mapping of Mixtures of Reactions on a Kohonen SOM

**Figure 2:**
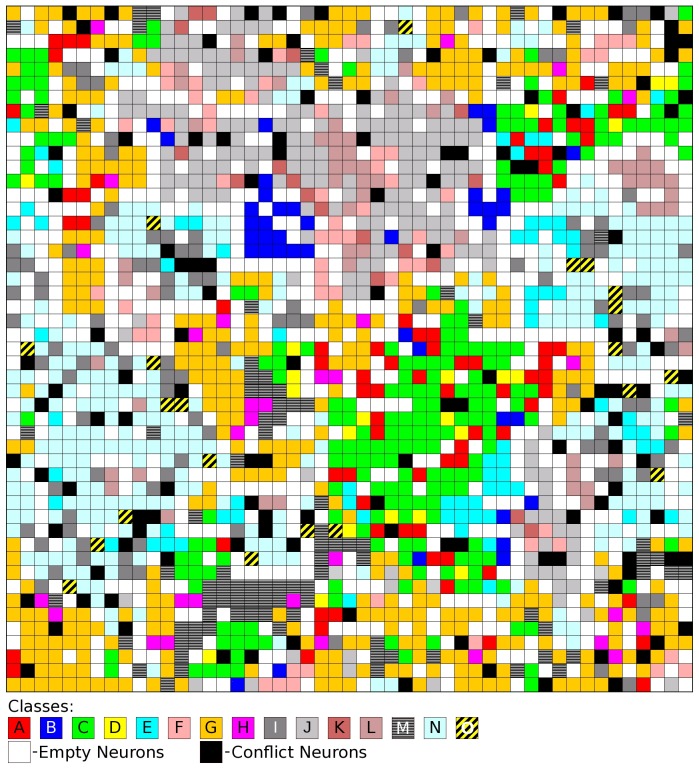
Toroidal surface of a 49×49 Kohonen SOM trained with 8280 mixtures of two photochemical reactions encoded by the ^1^H NMR descriptor. After the training, each neuron was colored according to the reaction mixtures of the training set that are mapped onto it. The colors correspond to the classes in Table 1. Black neurons correspond to conflicts.

Kohonen SOMs of size 49×49 were trained using the NMR reaction descriptors for mixtures in the training set. In the learning procedure, the network made no use of the information related to mixture classes. After the training, each neuron of the surface was assigned to a mixture class (one of the 15 possible combinations of two reactions from six types). Figure 2 shows a Kohonen SOM of size 49×49 trained with 8280 mixtures corresponding to the training set of partition 1.

**Figure 3:**
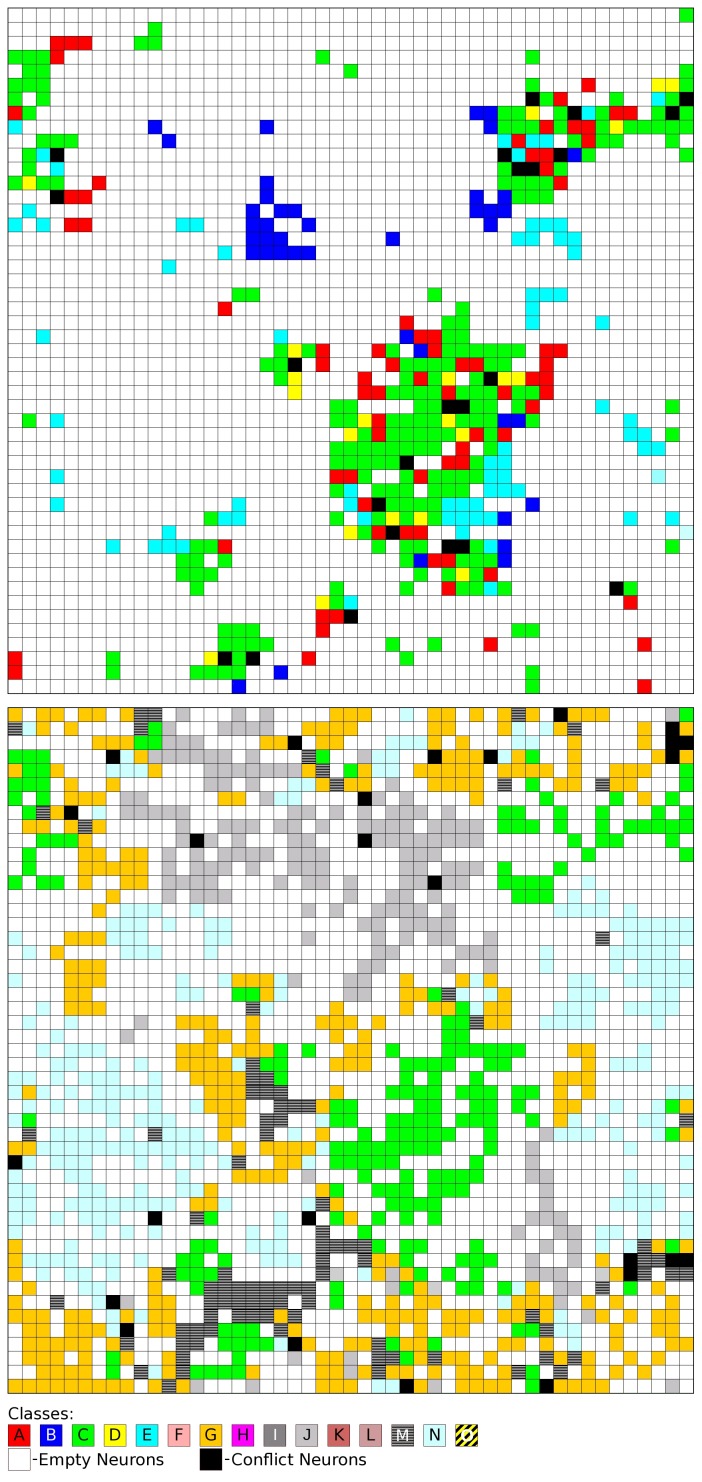
The same map of Figure 2, with two different filters applied: top – only colored neurons belonging to mixtures of classes A, B, C, D, and E; bottom – only colored neurons belonging to mixtures of classes C, G, J, M, and N. The colors correspond to the classes in Table 1. Black neurons correspond to conflicts between these classes and white neurons correspond to empty neurons or neurons belonging to other classes.

The results show a trend for some classes of mixtures to cluster, namely class B, class C, class J, class L, class M and class N (see Table 1 for detailed information concerning the types of reactions in each class of mixtures). The 15 classes of mixtures correspond to combinations of two reactions from six different types. In fact, classes of mixtures sharing one type of reaction tend to be mapped on the same region of the map. This is illustrated in Figure 3, which results from applying two different filters to Figure 2. In the first map, only neurons were colored that correspond to mixture classes A, B, C, D, or E (in all these mixtures is present a reaction of type [3+2] photocycloaddition of azirines to C = C). These mixtures concentrate on certain regions of the map and are not well separated from each other (the exception is class B). The second map only shows colored neurons of mixture classes C, G, J, M, or N (these are the classes sharing a reaction of type [2+2] photocycloaddition of C = C to C = C). These mixtures are much spread through the map (because of the large number of reactions) and are well distinguished from each other. The two images illustrate how the overlap of mixture classes on the map corresponds to the overlap of types of reactions in the mixtures.

An individual SOM was able to consistently classify 80.6% of the reaction mixtures in the training set, and to correctly predict 71.1% of the test set (Table 3). Improvement in accuracy was achieved with ensembles of five and ten SOMs. Correct predictions were obtained for 86.7% and 89% of the training set, and 77.4% and 79.6% of the test set using ensembles of five and ten SOMs, respectively.

**Table 3:** Classification of mixtures of reactions (mixtures of two reactions) by Kohonen SOMs and Counter-Propagation Neural Networks of dimension 49×49.

Data sets*		% Correct predictions					
		Best ind.		Ensemble of five		Ensemble of ten	
		SOM	CPNN	SOM	CPNN	SOM	CPNN
Partition	Training	80.6	61.3	86.7	73.0	89.0	75.6
1	Test	71.1	57.7	77.4	69.1	79.6	71.8
Partition	Training	82.9	68.4	89.4	77.4	91.4	78.6
2	Test	52.6	47.2	59.4	57.2	62.6	57.5

*Partition 1–8280 and 4141 mixtures of reactions in training and test set, respectively; Partition 2–7578 and 593 mixtures of reactions in training and test set, respectively.

Then, experiments were performed using partition 2, with lower similarities between training and test sets. With partition 1, all mixtures are different, but the same reaction is often present in a mixture of the training and in a mixture of the test set (combined with another reaction). Differently, with partition 2 no reaction in mixtures of the test set was present in a mixture of the training set. Not surprisingly, the prediction accuracy decreased considerably – an ensemble of ten SOMs was able to correctly classify 62.3% of the mixtures in the test set.

### Mapping of Mixtures of Reactions on a CPNN

CPNN process input data similarly to Kohonen SOM, but uses a different mechanism for producing classifications. A reaction type is identified in the mixture of the reactions if the activated neuron exhibits a high value for the output weight corresponding to that reaction type. Based on the six-values output, a mixture class is predicted if two and only two of the output values are higher than 0.5 (the mixture class corresponding to the combination of those two reaction types). Otherwise, the mixture is classified as undecided.

**Figure 4:**
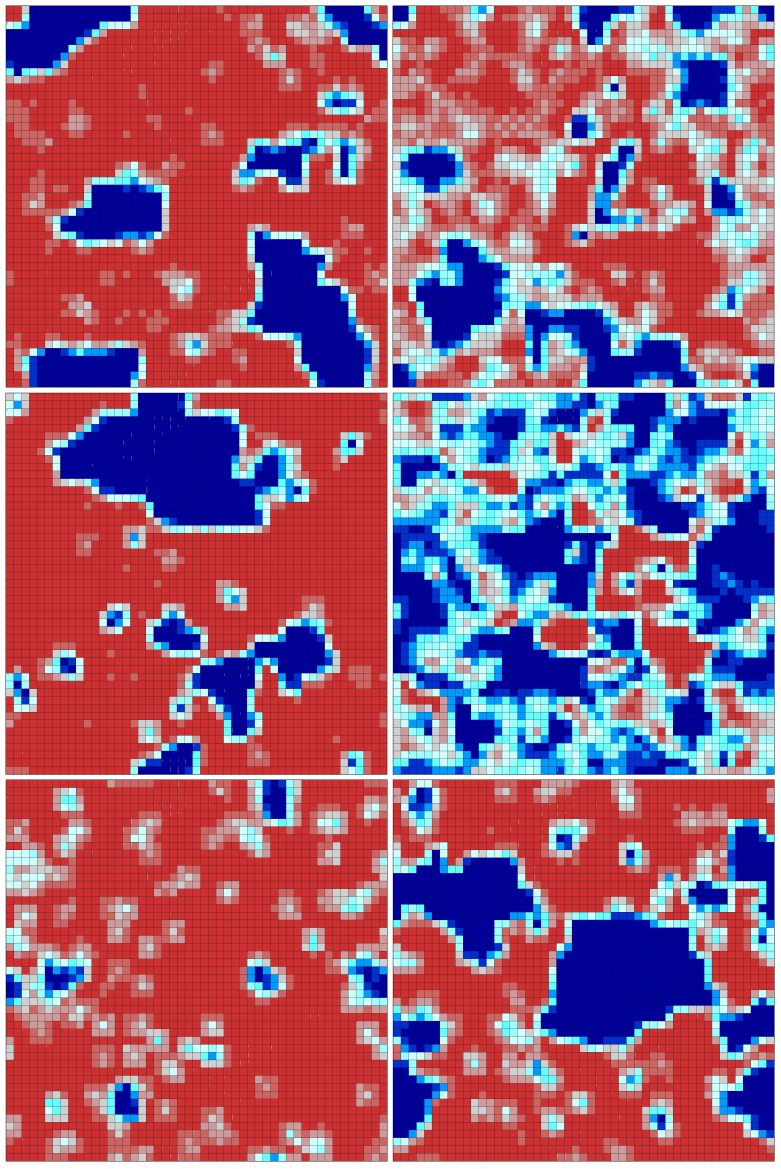
Representation of the six output layers of a 49×49 CPNN trained with 7578 mixtures of two reactions. High values of the weights in each output layer are represented by blue, and low values by red. Output layers corresponding to the following reaction types, from left to right: First row – [3+2] photocycloaddition of azirines to C = C and [2+2] photocycloaddition of C = C to C = O reaction types. Second row – [4+2] and [4+4] photocycloaddition of olefins to carbon-only aromatic rings, and [2+2] photocycloaddition of C = C to C = C reaction types. Third row – [3+2] photocycloaddition of s-triazolo[4,3-b]pyridazine to C = C and [2+2] photocycloaddition of C = C to C = S reaction types.

Figure 4 shows the six output layers (corresponding to the six possible types of reactions in the mixtures) of a 49×49 CPNN trained with 7578 mixtures (partition 2). High values of the weights at each output layer are represented by blue, and low values by red. It can be seen that mixtures including reactions from certain types cluster in typical regions while other types are more spread on the map and not so dominant in mixtures. The inspection of the six output layers reveals some correlation between the number of blue neurons in a layer, and the number of reactions of that type. For example, the most populated type of reaction in the data set is the [2+2] photocycloaddition of C = C to C = C with 73 reactions – it corresponds to output layer 4 with large regions of blue neurons. In fact, 4766 out of the 7578 mixtures in the training set (∼63%) include this type of reaction. In the opposite side, output layer 5 is mostly red and the corresponding reaction type ([3+2] photocycloaddition of s-triazolo[4,3-b]pyridazine to C = C) is the least populated reaction type with only 10 reactions, present in 1063 mixtures (∼14% of the training set).

CPNN did not yield superior predictions to Kohonen SOMs (Table 3). For partition 1 an ensemble of ten CPNNs were only able to correctly classify 75.6% and 71.8% of the mixtures of the training and test set respectively. The prediction accuracy for the test set decreased in partition 2 to 57.5%. It is important to point out that in CPNNs, with six output layers, a class is only assigned to a mixture when two and only two of the output values are higher than 0.5 (the mixture class corresponding to the combination of those two reaction types). If such an assignment is not possible, the mixture is predicted as undecided. This strict condition gives rise to a large number of mixtures with no class assigned. For example, the best individual CPNN does not assign ∼26% of the mixtures in the test set of partition 1. If only assigned mixtures are considered, the true classifications are 78%.

Kohonen SOM is an unsupervised learning method, and CPNN is semi-supervised. Both present the advantage of an easy visualization of the objects in a map, and reveal relationships between similarities of descriptors and classes. However, they are based on global comparisons of the descriptor profile, and are not expected to learn associations between classes and reduced numbers of specific descriptors. Such associations may well occur in the studied data set – some regions of the spectrum are likely to be more relevant than others. Therefore, experiments were next performed with a supervised learning method.

### Assignment of Reaction Types in Mixtures of Reactions by RFs

The results obtained with Random Forests are displayed in Table 4 for the two partitions. Predictions for training sets were from the internal cross validation obtained by out-of-bag (OOB) estimation. The accuracies of the predictions for partition 1 reached 99% both for OOB estimation of the training set and for the test set. A 10-fold cross-validation experiment was also performed with the training set of Partition 2. The obtained accuracy was similar to the OOB estimation and reached 99.5% of correct predictions. With a totally independent test set (partition 2) the accuracy of the predictions was 80%. RFs performed clearly better than the self-organizing maps. Table 5 shows the confusion matrix obtained for the test set of partition 2.

**Table 4:** Classification of mixtures of reactions (mixtures of two reactions) by Random Forests.

Data sets*		% Correct predictions
Partition	Training	99.2
1	Test	99.1
Partition	Training	99.6
2	Test	80.3

*Partition 1–8280 and 4141 mixtures of reactions in training and test set, respectively; Partition 2–7578 and 593 mixtures of reactions in training and test set, respectively.

**Table 5:** Confusion matrix for the classification of mixtures obtained by RF for the test set of partition 2.

	A	B	C	D	E	F	G	H	I	J	K	L	M	N	O	%
A	25	–	5	–	–	–	2	–	–	–	–	–	–	–	–	78.1
B	–	15	–	–	–	–	–	–	–	1	–	–	–	–	–	93.8
C	5	–	60	–	–	–	3	–	–	–	–	–	–	–	–	88.2
D	–	–	–	8	–	–	–	–	–	–	–	–	–	–	–	100.0
E	1	–	7	–	10	–	–	–	–	–	–	–	–	2	–	50.0
F	–	–	–	–	–	28	1	–	–	3	–	–	–	–	–	87.5
G	–	–	1	–	–	–	135	–	–	–	–	–	–	–	–	99.3
H	–	–	–	–	–	–	–	16	–	–	–	–	–	–	–	100.0
I	–	–	–	–	–	–	16	–	21	–	–	–	–	3	–	52.5
J	–	–	–	–	–	8	1	–	–	59	–	–	–	–	–	86.8
K	–	–	–	–	–	–	–	–	–	–	8	–	–	–	–	100.0
L	–	–	–	–	–	2	–	–	–	7	–	11	–	–	–	55.0
M	–	–	–	–	–	–	–	–	–	–	–	–	34	–	–	100
N	–	–	3	–	–	–	34	–	7	–	–	–	–	41	–	48.2
O	–	–	–	–	–	–	–	–	–	–	–	–	5	–	5	50.0

The confusion matrix shows a high prediction accuracy not only for the most populated classes like class C, G and J, but also for some less populated like classes D, H and K with 100% of correct classifications. The mixtures of classes E, I, L, N and O are the most difficult to classify. For them, true positives are only ca. 50% of the number of mixtures for these classes (last column of Table 5), although the counts of false positives are relatively low (inspection of Table 5 by columns). Classes E, I, L. N and O result from the combination of [2+2] photocycloadditions of C = C to C = S with the remaining five types of reactions, which indicates that the patterns of this reaction type, encoded in our “reaction spectrum”, is from all types of reactions the most difficult to learn – a consequence of a lack of hydrogen atoms bonded to the atoms of the reaction center in our data set. The difficulty to learn this type of reaction was also found in our previous studies [21] for the classification of reactions outside of mixtures. This approach cannot properly encode mixtures of reactions where the reactants and products have no hydrogen atoms bonded to the atoms of the reaction center. The prediction ability increase from 80% to 93% if the mentioned classes are not considered.

RF associate a probability to each prediction reflecting the proportion of votes obtained by the winning class. Table 6 presents the relationship between the prediction accuracy and the probability of the predictions.

**Table 6:** Relationship between the prediction accuracy and the probability associated to each prediction by RFs for test set of partition 2.

Classes^a^	Probability							
	No Selection		≥0.5		≥0.6		≥0.8	
	N.of Mixtures^b^	N. of Correct^c^	N.of Mixtures^b^	N. of Correct^c^	N.of Mixtures^b^	N. of Correct^c^	N.of Mixtures^b^	N. of Correct^c^
A (32)	31	25 (80.7)	12	11 (91.7)	4	4 (100.0)	2	2 (100.0)
B (16)	15	15 (100.0)	7	7 (100.0)	4	4 (100.0)	1	1 (100.0)
C (68)	76	60 (79.0)	52	50 (96.1)	41	41 (100.0)	14	14 (100.0)
D (8)	8	8 (100)	6	6 (100.0)	4	4 (100.0)	2	2 (100.0)
E (20)	10	10 (100)	3	3 (100.0)	3	3 (100.0)	–	–
F (32)	38	28 (73.7)	24	21 (87.5)	17	16 (94.1)	6	6 (100.0)
G (136)	192	135 (87.5)	130	115 (94.1)	91	88 (96.7)	37	37 (100.0)
H (16)	16	16 (100.0)	14	14 (100.0)	14	14 (100.0)	5	5 (100.0)
I (40)	28	21 (75.0)	17	15 (88.2)	10	8 (80.0)	–	–
J (68)	70	59 (84.3)	43	41 (95.4)	27	26 (96.3)	11	10 (90.9)
K (8)	8	8 (100.0)	8	8 (100.0)	7	7 (100.0)	6	6 (100.0)
L (20)	11	11 (100.0)	7	7 (100.0)	5	5 (100.0)	1	1 (100.0)
M (34)	39	34 (87.2)	30	30 (100.0)	28	28 (100.0)	14	14 (100.0)
N (85)	46	41 (89.1)	25	25 (100.0)	13	13 (100.0)	6	6 (100.0)
O (10)	5	5 (100.0)	5	5 (100.0)	3	3 (100.0)	2	2 (100.0)
Total	593	476 (80.3)	383	358 (93.5)	271	264 (97.4)	107	106 (99.1)

a Class labels and number of reactions in each class.

b Number of mixtures predicted to belong to each class.

c Number of true positives for each class and (in parenthesis) its percentage among the number of mixtures predicted to belong to that class.

The results support the use of the probability of each prediction as a measure of reliability of the class assignment. For the test set of the second partition, 383 mixtures out of 593 (65%) were predicted with probability higher than 0.5 and, from these, 358 (94%) were correctly classified. If we consider only mixtures predicted with probability higher than 0.6, the number of predicted mixtures decreases to 271 (46%) but the percentage of correctly classified mixtures (among these) increases to 97%. With a probability higher than 0.8, almost all mixtures of reactions were correctly classified (106 out of 107).

Filtering predictions by the RF probability also improves the results for the more problematic mixture classes E, I, L N, and O (Table 7). The percentage of true positive predictions among the mixtures of each class predicted with probability above 0.5 increased to 60%, 60%, 88%, 78%, and 100% respectively – Tables 5 and 6.

**Table 7:** Confusion matrix for the classification of mixtures with probability higher than 0.5 obtained by RF for the test set of partition 2.

	A	B	C	D	E	F	G	H	I	J	K	L	M	N	O	%
A	11	–	–	–	–	–	–	–	–	–	–	–	–	–	–	100.0
B	–	7	–	–	–	–	–	–	–	–	–	–	–	–	–	100.0
C	1	–	50	–	–	–	–	–	–	–	–	–	–	–	–	98.0
D	–	–	–	6	–	–	–	–	–	–	–	–	–	–	–	100.0
E	–	–	2	–	3	–	–	–	–	–	–	–	–	–	–	60.0
F	–	–	–	–	–	21	–	–	–	1	–	–	–	–	–	95.5
G	–	–	–	–	–	–	115	–	–	–	–	–	–	–	–	100.0
H	–	–	–	–	–	–	–	14	–	–	–	–	–	–	–	100.0
I	–	–	–	–	–	–	–	–	15	–	–	–	–	–	–	60.0
J	–	–	–	–	–	3	–	–	–	41	–	–	–	–	–	93.2
K	–	–	–	–	–	–	–	–	–	–	8	–	–	–	–	100.0
L	–	–	–	–	–	–	–	–	–	1	–	7	–	–	–	87.5
M	–	–	–	–	–	–	–	–	–	–	–	–	30	–	–	100.0
N	–	–	–	–	–	–	5	–	2	–	–	–	–	25	–	78.1
O	–	–	–	–	–	–	–	–	–	–	–	–	–	–	5	100.0

The best RF model developed with partition 1 was further validated using the y-randomization technique. The model was retrained using a modified training set where the Y-column values – the column corresponding to the classification of the mixtures - was scrambled and the descriptor matrix was kept unchanged. Scrambling was performed 5 times. Each randomized model was used to make predictions for the test set. A considerable decrease in the % of correct predictions in comparison with the non-randomized model was observed for the five random models (% of correct predictions: 14.5–15.7%) which supports the reliability and robustness of the original model.

To check the impact of the random partition in training and test sets, five random alternative partitions to partition 1, with the same sizes of the training and test sets, were used to train RF models. The results, both for training sets (OOB estimation) and test sets were similar to those of Table 4 for partition 1 (99.1% for the original test set partition and a range of 99.2–99.5% of correct predictions for the new five randomly selected partitions).

In order to better simulate realistic situations and possible experimental conditions, the RF model trained with partition 1 was further validated using more challenging test sets, generated with partial conversion of the reactants into products and different ratios of the two reactions in the mixture. The accuracy of the predictions for the test set of partition 1 with 70%, 80% and 90% simulated reaction yields (for both reactions of the mixture) was 81.7%, 96.4% and 98.8% respectively. These compare with 99.1% of correct predictions for the test set with full conversion (Table 4).

The test set of partition 1 was also re-used to simulate different ratios of the two reactions in a mixture and different normalizations of the spectra integration. A MIXTURE *i* was generated by the formula MIXTURE*_i_* = NORM*(RATIO * A_i_+B_i_), where A_i_ and B_i_ are the reactions of the mixture, RATIO took values 2 and 5, and NORM took random values between 0.2 and 1.0. Table 8 shows how the RF model developed with the training set of partition 1 (where NORM and RATIO were always 1) predicted the new test set (consisting of 8,282 mixtures).

**Table 8:** Impact of the ratio of the two reactions in the mixture and the integration normalization on the % of correct predictions.^a^

RATIO A_i_/B_i_	% Correct Predictions	
	NORM = 1	0.2≤NORM≤1
1 (Table 3)	99.1	–
2	96.2	75.3
5	82.7	70.6

a The same mixtures of the test set of partition 1 were used, but with different ratios between the two reactions, and different normalization factors in the spectra integration.

Finally, the test set was simulated with simultaneous random variation of the three parameters – yields, NORM (range 0.2–1.0) and RATIO (range 1–4). The percentage of correct predictions for test sets with reaction yields ranges of 50–100%, 60–100% and 70–100% were 62, 65 and 68% respectively.

A relationship between the probability of the RF predictions and the prediction accuracy was observed again, for the most challenging test set – yields (range 50%−100%), NORM (range 0.2–1.0) and RATIO (range 1–4) – Table 9.

**Table 9:** Relationship between the prediction accuracy and the probability associated to each prediction by RFs for the test set of partition 1 simulated with simultaneous random variation of the three parameters – yields (range 50–100%), NORM (range 0.2–1.0) and RATIO (range 1–4).

Classes^a^	Probability							
	No Selection		≥0.5		≥0.6		≥0.8	
	N.of Mixtures^b^	N. of Correct^c^	N.of Mixtures^b^	N. of Correct^c^	N.of Mixtures^b^	N. of Correct^c^	N.of Mixtures^b^	N. of Correct^c^
A (414)	179	178 (99.4)	141	141 (100)	95	95 (100)	17	17 (100)
B (266)	79	75 (94.9)	25	25 (100)	8	8 (100)	2	2 (100)
C (974)	1108	751 (67.8)	863	642 (74.4)	693	556 (80.2)	322	291 (90.4)
D (134)	62	61 (98.4)	44	44 (100)	36	36 (100)	9	9 (100)
E (360)	163	146 (89.6)	96	94 (97.9)	57	57 (100)	6	6 (100)
F (412)	116	106 (91.4)	68	66 (97.1)	48	48 (100)	11	11 (100)
G (1510)	3327	1476(44.4)	2600	1367(52.6)	2208	1269(57.5)	1250	895 (71.6)
H (206)	89	89 (100)	74	74 (100)	61	61 (100)	36	36 (100)
I (558)	224	221 (98.7)	153	153 (100)	96	96 (100)	21	21 (100)
J (972)	670	477 (71.2)	417	325 (77.9)	313	256 (81.8)	121	108 (89.3)
K ()134	36	26 (72.2)	29	22 (75.9)	23	21 (91.3)	9	9 (100)
L (360)	122	115 (94.3)	64	64 (100)	37	37 (100)	7	7 (100)
M (488)	449	288 (64.1)	352	243 (69)	283	200 (70.7)	196	155 (79.1)
N (1314)	1586	1046 (66)	1230	880 (71.5)	964	730 (75.7)	434	353 (81.3)
O (180)	72	67 (93.1)	52	51 (98.1)	33	33 (100)	11	11 (100)
Total	8282	5122(61.8)	6208	4191(67.5)	4955	3503(70.7)	2452	1931(78.8)

a Class labels and number of reactions in each class.

b Number of mixtures predicted to belong to each class.

c Number of true positives for each class and (in parenthesis) its percentage among the number of mixtures predicted to belong to that class.

In this test set, 6208 mixtures out of 8282 (75%) were predicted with probability higher than 0.5 and, from these, 4191 (68%) were correctly classified. If we consider only mixtures predicted with probability higher than 0.8, the number of predicted reactions decreases to 2452 (30%) but the percentage of correctly classified mixtures (among these) increases to 79%. It is to point out the results for the more difficult class G. From the 3327 mixtures classified as G, only 1476 (44%) were correctly classified, but the percentage increases to 72% among mixtures predicted with probability higher than 0.8.

The results clearly show that the model learns the key patterns of NMR signals corresponding to classes of reactions in the mixtures and are reasonably capable of classifying new cases involving partial conversion of reactants, different ratios between reactions and different normalization of integrations, even without any re-parameterization of the initial model.

Clearly, if these more demanding situations are included in the training set, the ability to predict the test set are improved. For example, in an experiment where both training and test sets are simulated with simultaneous random variation of the three parameters – yields (range 25%–100%), NORM (range 0.2–1.0) and RATIO (range 1–7) – a RF correctly predicted 96% of the mixtures, which reinforces the conclusion that the model learns the classes of reactions by the presence of key patterns of NMR signals.

### Conclusions

This study demonstrates the possibility of applying machine learning methods to automatically identify types of co-occurring chemical reactions from the differences between the ^1^H NMR spectra of reactants and products. These results also illustrate the usefulness of SPINUS predictions of NMR data in that context, for the generation of training sets.

The fact that a supervised learning method yielded significantly better predictions suggests that changes in very specific ranges of the ^1^H NMR spectra are markers of reaction types. The extremely high percentages of correct predictions for the test set of partition 1 with supervised learning, and for the random forest OOB estimation within training sets of both partitions, indicate that the same should happen for individual reactions.

In most practical situations, a reaction is accompanied by side reactions, and can proceed to different yields, which would require that the NMR interpretation system is able to identify reaction types even in the presence of a complex mixture of reactions with different conversions. Experiments simulating mixtures of reactions with a diversity of product yields, different proportions of reactions, and different normalization of integrations corroborated this possibility.

This study relies on ^1^H NMR spectra, and is therefore limited by the availability of hydrogen atoms in the neighborhood of the reaction center and by the sensitivity of their chemical shifts to the changes resulting from the reactions. But, in principle, the method can be used with other types of spectra, e.g., ^13^C NMR or IR. It must be emphasized that this approach does not require structural information on the reactions participants – it performs “reaction elucidation” without structure elucidation of the molecules in the mixtures.
